# Drug resistance mutations to integrase inhibitors, proteinase, and reverse transcriptase inhibitors in newly diagnosed HIV-1 infections in Hebei province, China, 2018–2022

**DOI:** 10.3389/fcimb.2025.1510916

**Published:** 2025-02-24

**Authors:** Xinli Lu, Yan Li, Meng Liu, Yingying Wang, Ning An, Dandan Sun, Qi Li

**Affiliations:** Department of AIDS Research, Hebei Key Laboratory of Pathogen and Epidemiology of Infectious Disease, Hebei Provincial Center for Disease Control and Prevention, Shijiazhuang, Hebei, China

**Keywords:** HIV-1, primary drug resistance, integrase strand transfer inhibitors, network, MSM

## Abstract

**Background:**

HIV-1 protease (PR)-reverse transcriptase (RT) inhibitors as national free antiretroviral drugs have been used for 20 years. Integrase strand transfer inhibitors (INSTIs) have been conditionally used as a component of HIV/AIDS treatment regimens in recent years. However, the systematic investigation on the changes in primary drug resistance (PDR) in Hebei province, China was limited.

**Methods:**

A continuous cross-sectional investigation on HIV-1 PDR was conducted, integrating detection of drug resistance genotype, molecular network, and statistical analysis.

**Results:**

The overall prevalence of PDR was 8.3%, with 77 of 925 samples showing different levels of resistance to INSTIs (1.9%), protease inhibitors (PIs, 0.2%), nucleoside reverse transcriptase inhibitors (NRTIs, 1.2%), and non-NRTIs (NNRTIs, 5.2%). In the PR-RT gene coding region, E138EK/G was the most common (1.6%), followed by K103N (1.4%), G190GE/A/S (0.6%), K101E (0.5%), A98G (0.4%), and T215I/TS (0.3%), associated with the low- to high-level resistance to doravirine (DOR), efavirenz (EFV), etravirine (ETR), nevirapine (NVP), rilpivirine (RPV), and zidovudine (AZT). In the INSTI gene coding region, six mutations were identified, namely, four major mutations (P145PS, Q148QH, Y143S, and T66A) and two accessory mutations (S153SF and G163GRS/EK). Of these mutations, the most frequent INSTI mutations were S153SF (0.6%) and G163GRS/EK (0.6%), followed by P145PS (0.2%), Y143S (0.2%), Q148QH (0.1%), and T66A (0.1%). G163GRS/EK, P145PS, Y143S, and T66A were associated with the resistance to elvitegravir (EVG) and raltegravir (RAL). S153SF and Q148QH were mainly related to the resistance to dolutegravir (DTG), bictegravir (BIC), and caboteravir (CAB). Furthermore, 30 resistant sequences were circulating in 16 transmission networks with HIV-1 DR mutations (DRMs), accounting for 62.5% of 77 total participants with DRMs. Multivariable analysis showed that those who had CRF07_BC had 1.79 times greater odds of PDR compared with participants with CRF01_AE. Compared to participants with volunteer blood donor, those with voluntary consultation and testing had 0.27 times greater odds of PDR.

**Conclusions:**

The overall prevalence of HIV-1 PDR in Hebei is high, belonging to a moderate resistant level (5.0%–15.0%). It is necessary for us to strengthen the effective surveillance of PDR among treatment-naive patients, and we should adjust the treatment plan according to the results of PDR surveillance.

## 1 Introduction

Human immunodeficiency virus (HIV) has rapidly spread across the globe via sexual contact, blood transfusion, and mother-to-child transmission since the first acquired immune deficiency syndrome (AIDS) patient in 1981. Globally, there were 39.9 million people living with HIV (PLWH) in 2023 (UNAIDS). Highly active antiretroviral therapy (HAART) has obviously decreased the mortality of HIV/AIDS patients and prolonged their lives in the past decades (UNAIDS; Bershteyn et al., 2022). However, there were still 1.3 million new HIV infections, and 630,000 people died of AIDS-related illnesses in 2023 (UNAIDS). The main reason is that the occurrence of HIV drug resistance (DR) seriously reduces the effect of HAART, being one of the main obstacles to achieving the goal of ending the AIDS epidemic by 2030. In particular, two of three 95% targets are closely associated with the effects of HAART.

In China, marvelous successes in HIV/AIDS prevention and control have been achieved in the past four decades (Xu et al., 2021; Dou et al., 2023). Previously, UNAIDS estimated that the number of HIV/AIDS cases would reach 10 million by the end of 2010 (Kaufman and Jing, 2002); however, there were 1.29 million PLWH actually reported across China until 2023. At the country level in China, the reported rate and mortality of HIV-infected individuals have indicated a long-term slow increasing trend, but they started to decrease in 2018 (Dou et al., 2023). The “Four Frees and One Care” policy played a critical role in China’s achievements (Zhao et al., 2023): 92.8% HAART coverage and 97.0% HAART success (viral load ≦ 1,000 copies/mL) rate have been achieved as of 2022. However, there is still approximately 3.0% of PLWH who have experienced ART failure, and HIV DR mutation (DRM) and low-level viremia were the main factors of ART failure and death (Lucas et al., 2018).

China has five of six categories of antiretroviral drugs (ARDs) used globally. Of them, nucleoside reverse transcriptase inhibitors (NRTIs), nonnucleoside reverse transcriptase inhibitors (NNRTIs), and partial proteinase inhibitors (PIs) such as lopinavir (LPV)/r were included in national free antiretroviral regimens; however, all HIV/AIDS patients have to take integrase strand transfer inhibitor (INSTI) medication at their own expense in China. The numerous researches on DR paid more attention to the resistance to NRTIs, NNRTIs, and PIs, especially acquired drug resistance (ADR) in treatment-failure patients. Of 28,510 patients receiving treatment, 51.2% had DRMs in 2021 and the ADR prevalence of NNRTIs, NRTIs, and LPV/r was 48.8%, 27.4%, and 1.8%, respectively (Zhao et al., 2023). Among treatment-naïve HIV individuals, the overall resistance rate increased from 2.6% in 2004 to 6.9% in 2021 to 2022, with a significant elevation trend (*p* < 0.01) (Liu et al., 2023). INSTIs have been permitted to use in China in recent several years. The systematic investigation on primary drug resistance (PDR) to INSTIs in China is few. Therefore, we should pay more attention to the surveillance of HIV-1 PDR in order to end the AIDS epidemic by 2030. The present study analyzed HIV-1 DRMs to INSTIs, NRTIs, NNRTIs, and PIs among treatment-naive HIV-1 individuals and their transmission networks.

## 2 Materials and methods

### 2.1 Ethics statement

Written informed consents were obtained from all adults ≥ 18 years and children’s guardians ahead of sample collection. The current study was approved by the Medical Ethics Committee of Hebei provincial center for disease control and prevention [No. IRB(S)2020-031]. All experimental methods, study procedures, and study items were performed in accordance with approved regulations and guidelines.

### 2.2 Study population

In this study, we collected 1,008 blood samples from newly diagnosed HIV-1 individuals between 2018 and 2022. These participants’ baseline data, such as age, gender, nationality, infection routes, first CD4 cell count, marital status, education level, occupation, sample source, etc., were obtained from the HIV database of the National Center for AIDS/STD Control and Prevention. For geographic location, the participants were grouped into four regions according to the Hukou, including Jibei, Jizhong, Jidong, and Jinan. The participants should meet the following criteria: (a) age ≥15 years; (b) have sample collection time; (c) naïve treatment; and (d) newly diagnosed HIV-1 infection. A cross-sectional investigation on HIV-1 PDR was conducted, integrating detection of DR genotype, molecular networks, and statistical analysis.

### 2.3 HIV-1 RNA extraction, amplification, and subtype confirmation

Based on the method previously reported by us (Lu et al., 2024), HIV-1 RNA was extracted from 200 µL of blood plasma using the Roche MagNa pure total RNA kit (Qiagen, Valencia, CA, USA) and the *pol* gene fragment (HXB2:2068–5221) of HIV-1 RNA was amplified using the previous primers (Lu et al., 2024) and cycling conditions (Lu et al., 2017). Beijing Biomed Gene Technology Co., Ltd. (Beijing, China) carried out sequence determination using Sanger’s method. HIV-1 subtypes were preliminarily inferred using the online HIV Blast Microsoft (https://www.hiv.lanl.gov/content/sequence/BASIC_BLAST/basic_blast.html), followed by the phylogenetic analysis based on *pol* gene sequences using MEGA 7.0 and confirmed using the online REGA HIV-1 Subtyping Tool-Version 3.0 (http://dbpartners.stanford.edu:8080/RegaSubtyping/stanford-hiv/typingtool/).

### 2.4 HIV-1 resistance mutations and molecular networks

HIV-1 *pol* sequences containing proteinase, reverse transcriptase, and integrase gene coding regions were submitted to the Stanford University HIV Drug Resistance Database (http://hivdb.stanford.edu/). DRMs to PIs, NRTIs, NNRTIs, and INSTIs were analyzed using the HIVDB algorithm version 9.5.1. Moreover, the resistance level was classified into four categories according to individual mutation score, namely, potential low-level resistance (10–14), low-level resistance (15–29), intermediate-level resistance (30–59), and high-level resistance (≥60). Molecular transmission networks were constructed based on the study *pol* sequences. Pairwise genetic distances were calculated using HYPHY2.2.4 with a Tamura-Nei 93 (TN93) model. We selected a genetic distance threshold of 0.015 substitutions/site to construct networks because this threshold is consistent with recent and rapid transmission. Molecular transmission networks were visualized using HIV-Trace-1.5.0.

### 2.5 Statistical analysis

Statistical analysis was implemented using SPSS 23.0 (SPSS Inc., Chicago, IL, USA). Means and frequencies were used to summarize demographic data. Differences in categorical variables were analyzed using the chi-square (χ^2^) test. The multivariable logistic regression was carried out in order to identify the association between factors of interest and the presence of PDR. Statistical significance was defined as *p* < 0.05 for all tests. Spearman’s method was utilized to analyze the epidemic trend of HIV-1 PDR with a *p*-value of 0.05 (χ^2^ trend): *r* < 0 denotes a negative correlation, and *r* > 0 denotes a positive correlation.

## 3 Results

### 3.1 Basic characteristics

In total, 1,008 participants were enrolled in this study. Of them, 925 HIV-1 *pol* sequences (HXB2:2068–5221) were obtained, and the PCR positive rate was 91.8%. As shown in **Table 1**, men accounted for 97.4% (901/925) of the participants. Participants aged 25–49 accounted for 60.0% (555/925). The breakdown of participant occupation was as follows: farmer, 28.1% (260/925); housework/freelance, 26.5% (245/925); service, 21.2% (196/925); cadre/staff/teacher, 15.7% (145/925); and student, 8.5% (79/925). A total of 46.7% (432/925) were unmarried. Han ethnicity accounted for 939% (869/925). A total of 67.1% (621/925) had an educational level of high school or below, followed by college or above (32.9%, 304/925). A total of 55.0% (509/925) had CD4 cell counts > 200 cells/μL. Men who have sex with men (MSM) accounted for 93.0% (860/925). Among four sample sources, voluntary consultation and testing (VCT) was the most frequent, accounting for 62.2% (575/925), followed by visit test (23.2%, 215/925).

**Table 1:** Demographic characteristics of HIV-1-infected participants enrolled in this study.

Variable	Number of participants	NFLGs *N* (%)	Number of DR				*χ* ^2^	*p*
			PI	NRTI	NNRTI	INSTI		
Total	1,008	925 (91.8)	2	11	48	18		
Gender								
Male	984	901 (91.6)	2	11	48	18		
Female	24	24 (100.0)	0	0	0	0		
Age							5.510	0.439
15–24	208	192 (92.3)	0	1	11	7		
25–49	613	555 (90.5)	2	9	34	9		
≥50	187	178 (95.2)	0	1	3	2		
Occupation							8.805	0.734
Service	211	196 (92.9)	0	2	16	6		
Student	83	79 (95.2)	0	0	6	2		
Housework/freelance	265	245 (92.3)	0	3	8	4		
Cadre/staff/teacher	159	145 (91.2)	1	3	5	1		
Farmer	290	260 (89.7)	1	3	13	5		
Marital status							11.973	0.034
Divorced/widowed	142	126 (88.7)	0	1	2	1		
Unmarried	463	432 (93.3)	0	2	30	12		
Married/cohabitation	403	367 (91.1)	2	8	16	5		
Nationality							1.763	0.693
Han	943	869 (92.2)	2	11	45	16		
Ethnic Minority	65	56 (86.2)	0	0	3	2		
Educational level							5.862	0.408
Junior school or below	426	397 (93.2)	0	6	21	7		
High school	249	224 (90.0)	1	0	10	5		
College or above	333	304 (91.3)	1	5	17	6		
Initial CD4 counts (cells/μL)							13.704	0.014
≤200	180	170 (94.4)	2	3	9	2		
201–500	550	509 (92.5)	0	8	19	9		
>500	278	246 (88.5)	0	0	20	7		
Infection route							3.146	1.000
MSM	943	860 (91.2)	2	11	47	18		
Heterosexual	65	65 (100.0)	0	0	1	0		
Sample source							6.375	0.708
VCT	620	575 (92.7)	2	4	27	10		
Visit test	237	215 (90.7)	0	3	11	4		
VBD	37	31 (83.8)	0	2	4	0		
Special survey	114	104 (91.2)	0	2	6	4		
Subtype							19.748	0.627
CRF01_AE		454	1	5	18	12		
CRF07_BC		302	1	5	20	6		
URFs		57	0	1	0	0		
B		47	0	0	3	0		
CRF55_01B		28	0	0	5	0		
CRF65_cpx		7	0	0	1	0		
CRF68_01B		5	0	0	1	0		

NFLGs, near full-length *pol* sequences; DR, drug resistance; MSM, men who have sex with men; VCT, voluntary consultation and testing; VBD, volunteer blood donor.

### 3.2 DRMs to four categories of inhibitors

There were 77 (8.3%) of 925 study participants who had DRMs. Detailed DRMs are listed in **Table 2**. There were the most locations of gene mutations in 2020 during the five years, including PI-, NRTI-, NNRTI and INSTI-DRMs. However, no PI-resistant mutation was found in 2018, 2019, 2021, and 2022, and NRTI-DRM was also not found in 2018 and 2022, respectively. HIV-1 gene mutations resistant to ARDs were found each year between 2018 and 2022 in the NNRTI and INSTI gene coding regions, respectively. Eleven of 77 participants presented multiple drug resistance: in 2019, one participant (0.1%, 1/925) had two-point DRMs in the NNRTI gene coding region; in 2020, two participants (0.2%, 2/925) had double-point DRMs in the PI gene coding region, one participant (0.1%, 1/925) had three-point DRMs in the NRTI gene coding region, and four participants (0.4%, 4/925) had double (two cases) or four (two cases)-point DRMs in the NNRTI gene coding region; in 2022, two participants (0.2%, 2/925) carried a double-class PDR mutation pattern (NNRTI + INSTI), and another participant carried three mutations (S68SG, L74M, and A98G), but both S68SG and L74M cannot cause drug resistance.

**Table 2:** Distribution of HIV-1 drug resistance mutations in Hebei, 2018–2022.

DR mutations	2018 (*N* = 128)		2019 (*N* = 147)		2020 (*N* = 211)		2021 (*N* = 109)		2022 (*N* = 330)		Total (*N* = 925)
	DR cases (%)	ARDs	DR cases (%)	ARDs	DR cases (%)	ARDs	DR cases (%)	ARDs	DR cases (%)	ARDs	
Total	8 (6.3)		14 (9.5)		25 (11.8)		9 (8.3)		21 (6.4)		77 (8.3)
**PI-mutation**					**2 (0.9)**						**2 (0.2)**
F53L/I54L	–	–	–	–	1 (0.5)	ATV/LPV/DRV/r(L)	–	–	–	–	1 (0.1)
N88NT/L90LM	–	–	–	–	1 (0.5)	ATV/r(I);LPV/r(L)	–	–	–	–	1 (0.1)
**NRTI-mutation**			**1 (0.7)**		**5 (2.4)**		**1 (0.9)**		**4 (1.2)**		**11 (1.2)**
T215I	–	–	1 (0.7)	AZT(L)	–	–	–	–	1 (0.3)	AZT(L)	2 (0.2)
S68De/D67Del/K70KQ	–	–	–	–	1 (0.5)	ABC/TDF(H);AZT/FTC/3TC(I)	–	–	–	–	1 (0.1)
M184V	–	–	–	–	1 (0.5)	ABC(L);FTC/3TC(H)	–	–	–	–	1 (0.1)
T215TS	–	–	–	–	–	–	1 (0.9)	AZT(L)	–	–	1 (0.1)
L210LW	-	-	-	-	2 (0.9 )	AZT/D4T(L)	-	-	-	-	2 (0.2)
K65KN	-	-	-	-	1 (0.5)	ABC/DDI/D4T/TDF(I);FTC/3TC(L)	-	-	-	-	1 (0.1)
L74LI	–	–	–	–	–	–	–	–	1 (0.3)	ABC(L)	1 (0.1)
D67DN	–	–	–	–	–	–	–	–	2 (0.6)	AZT(L)	2 (0.2)
**NNRTI-mutation**	**5 (3.9)**		**9 (6.1)**		**15 (7.1)**		**7 (6.4)**		**12 (3.6)**		**48 (5.2)**
A98G	–	–	1 (0.7)	DOR/EFV/RPV(L);NVP(I)	–	–	1 (0.9)	DOR/EFV/RPV(L);NVP(I)	2 (0.6) ^a^	DOR/EFV/RPV(L);NVP(I)	4 (0.4)
E138EK/G	4 (3.1)	NVP/ETR/RPV(L)	2 (1.4)	NVP/ETR/RPV(L)	3 (1.4)	NVP/ETR/RPV(L)	1 (0.9)	NVP/ETR/RPV(L)	4 (1.2) ^C^	NVP/ETR/RPV(L)	14 (1.5)
G190GE/A	–	–	–	–	2 (0.9)	ETR(I),DOR/EFV/NVP/RPV(H)	1 (0.9)	ETR(I);DOR/EFV/NVP/RPV(H)	–	–	3 (0.3)
K101E	–	–	–	–	1 (0.5)	DOR/EFV/ETR(L);NVP/RPV(I)	–	–	–	–	1 (0.1)
K101E/E138K	–	–	1 (0.7)	DOR/EFV/ETR(L);NVP(I);RPV(H)	–	–	–	–	–	–	1 (0.1)
K101E/G190S/GE	–	–	–	–	1 (0.5)	ETR(I);DOR/EFV/NVP/RPV(H)	–	–	1 (0.3)	ETR(I);DOR/EFV/NVP/RPV(H)	2 (0.2)
K101E/G190S/V106VI	–	–	–	–	1 (0.5)	ETR(I);DOR/EFV/NVP/RPV(H)	–	–	–	–	1 (0.1)
K103N	–	–	3 (2.0)	NVP/EFV(H)	4 (1.9)	NVP/EFV(H)	3 (2.8)	NVP/EFV(H)	3 (0.9)^b,c^	NVP/EFV(H)	13 (1.4)
L100I	–	–	1 (0.7)	ETR/DOR(L);EFV/NVP/RPV(H)	1 (0.5)	DOR/ETR(L);EFV/NVP/RPV(H)	–	–	–	–	2 (0.2)
V108VI	–	–	–	–	1 (0.5)	NVP(L)	–	–	–	–	1 (0.1)
Y181C	1(0.8)	EFV/ETR/RPV(I); NVP(H)	–	–	–	–	–	–	–	–	1 (0.1)
Y188L	–	–	1 (0.7)	DOR/EFV/NVP/RPV(H)	–	–	1 (0.9)	DOR/EFV/NVP/RPV(H)	–	–	2 (0.2)
Y318YF	–	–	–	–	1 (0.5)	DOR(H); NVP(I)	–	–	–	–	1 (0.1)
K103KQ/K238KT	–	–	–	–	–	–	–	–	1 (0.3)	EFV/NVP(H)	1 (0.1)
H221Y									1 (0.3)	NVP/RPV(L)	1 (0.1)
**INSTI-mutation**	**3 (2.3)**		**4 (2.7)**		**3 (1.4)**		**1 (0.9)**		**7 (2.1)**		**18 (1.9)**
G163GRS/EK	2 (1.6)	EVG/RAL(L)			1 (0.5)	EVG/RAL(L)	1 (0.9)	EVG/RAL(L)	2 (0.6) ^c^	EVG/RAL(L)	6 (0.6)
P145PS ^d^	–	–	2 (1.4)	EVG(H)	–	–	–	–	–	–	2 (0.2)
Q148QH ^d^	–	–	–	–	1 (0.5)	BIC/DTG(L);CAB(I);EVG/RAL(H)	–	–	–	–	1 (0.1)
S153SF	1 (0.8)	BIC/CAB/DTG(L)	2 (1.4)	BIC/CAB/DTG(L)	–	–	–	–	3 (0.9) ^b^	BIC/CAB/DTG(L)	6 (0.6)
Y143S ^d^	–	–	–	–	1 (0.5)	RAL(H)	–	–	1 (0.3)	RAL(H)-	2 (0.2)
T66A ^d^	–	–	–	–	–	–	–	–	1 (0.3)	EVG(H)/RAL(L)	1 (0.1)

DR, drug resistance; ARD, antiretroviral drug; "-" denotes "none"; INSTIs, integrase strand-transfer inhibitors; H, high-level resistance; L, low-level resistance; I, intermediate-level resistance; PIs, proteinase inhibitor; NRTIs, nucleoside reverse transcriptase inhibitors; NNRTIs, non-nucleoside reverse transcriptase inhibitors; ^a^one participant carried three mutations (S68SG, L74M, and A98G) in 2022, but both S68SG and L74M cannot cause drug resistance; ^b^one participant carried two mutations (K103N and S153F) in 2022. ^c^one participant carried three mutations (E138EG, K103N and G163GR) in 2022. ^d^INSTI major mutations. Bold value denotes total mutation number of each ARD.

As shown in **Tables 2** and **3**, in the NRTI and PI gene coding regions, T215I/TS mutations were the most common, accounting for 0.3% (3/925), followed by L210LW (0.2%, 2/925) and D67DN (0.2%, 2/925), associated with the low-level resistance to zidovudine (AZT). The NNRTI gene coding region contained the most DRM positions: E138EK/G was the most common, accounting for 1.6% (12/925), followed by K103N (1.4%, 13/925), G190GE/A/S (0.6%, 6/925), K101E (0.5%, 5/925), A98G (0.4%, 4/925), L100I (0.2%, 2/925), and Y188L (0.2%, 2/925), associated with the different level of resistance to doravirine (DOR), efavirenz (EFV), etravirine (ETR), nevirapine (NVP), and rilpivirine (RPV).

**Table 3:** Frequency of HIV-1 primary drug resistance in 925 participants.

Gene coding region	Mutations	Frequency *N* (%)	Drugs	Frequency *N* (%)
PI	F53L	1 (0.1)	ATV/r	2 (0.2)
	I54L	1 (0.1)	LPV/r	2 (0.2)
	N88NT	1 (0.1)	DRV/r	1 (0.1)
	L90LM	1 (0.1)	–	–
NRTI	T215I/ TS	3 (0.3)	AZT	7 (0.8)
	L210LW	2 (0.2)	ABC	4 (0.4)
	D67DN	2 (0.2)	TDF	2 (0.2)
	M184V	1 (0.1)	FTC	3 (0.3)
	K65KN	1 (0.1)	3TC	3 (0.3)
	L74LI	1 (0.1)	D4T	3 (0.3)
	S68De	1 (0.1)	–	–
NNRTI	E138EK/G	15 (1.6)	DOR	17 (1.8)
	K103N	13 (1.4)	EFV	31 (3.4)
	G190GE/A/S	6 (0.6)	RPV	32 (3.5)
	K101E	5 (0.5)	NVP	48 (5.2)
	A98G	4 (0.4)	ETR	25 (2.7)
	Y188L	2 (0.2)	–	–
	L100I	2 (0.2)	–	–
	V108VI	1 (0.1)	–	–
	Y181C	1 (0.1)	–	–
	V106VI	1 (0.1)	–	–
	Y318YF	1 (0.1)	–	–
	K103KQ	1 (0.1)	–	–
	K238KT	1 (0.1)	–	–
	H221Y	1 (0.1)	–	–
INSTI	G163GRS/EK	6 (0.6)	EVG	10 (1.1)
	S153SF	6 (0.6)	RAL	10 (1.1)
	P145PS	2 (0.2)	BIC	7 (0.8)
	Y143S	2 (0.2)	DTG	7 (0.8)
	Q148QH	1 (0.1)	CAB	7 (0.8)
	T66A	1 (0.1)	–	–

"-" denotes "none"; INSTI, integrase strand-transfer inhibitor; PI, proteinase inhibitor; NRTI, nucleoside reverse transcriptase inhibitor; NNRTI, non-nucleoside reverse transcriptase inhibitor.

In the INSTI gene coding region, six mutations were identified, namely, four major mutations (P145PS, Q148QH, Y143S, and T66A) and two accessory mutations (S153SF and G163GRS/EK). Of them, the most frequent INSTI-DRMs were S153SF (0.6%, 6/925) and G163GRS/EK (0.6%, 6/925), followed by P145PS (0.2%, 2/925), Y143S (0.2%, 2/925), Q148QH (0.1%, 1/675), and T66A (0.1%, 1/675). G163GRS/EK, P145PS, Y143S, and T66A were associated with the resistance to elvitegravir (EVG) and raltegravir (RAL), and S153SF and Q148QH were mainly related to the resistance to dolutegravir (DTG), bictegravir (BIC), and cabotegravir (CAB).

It needs to be emphasized that the most frequent ARDs with a low- to high-level resistance were as follows: NVP (5.2%, 48/925), RPV (3.5%, 32/925), EFV (3.4%, 31/925), ETR (2.7%, 25/925), DOR (1.8%, 17/925), EVG (1.1%, 10/925), RAL (1.1%, 10/925), DTG (0.7%, 7/925), BIC (0.7%, 7/925), and CAB (0.7%, 7/925).

### 3.3 Epidemic pattern of resistant strains

**Figure 1:**
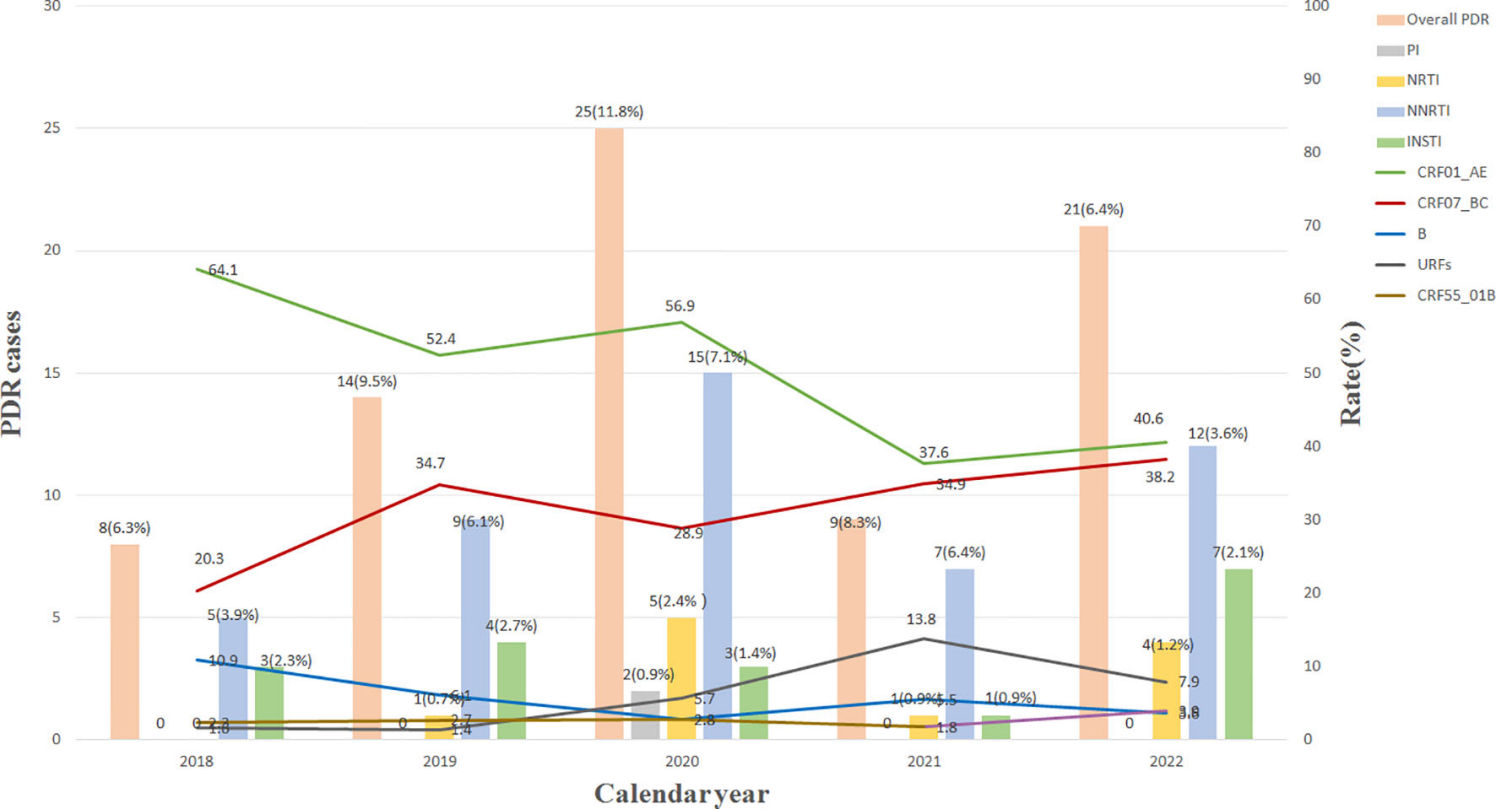
Changes of main HIV-1 subtypes and resistant strains from 2018 to 2022. INSTI, integrase strand-transfer inhibitor; PI, proteinase inhibitor; NRTI, nucleoside reverse transcriptase inhibitor; NNRTI, non-nucleoside reverse transcriptase inhibitor; URFs, unique recombinant forms; PDR, pretreatment drug resistance.

As shown in **Figure 1**, the overall prevalence of HIV-1 PDR increased from 6.3% (8/128) in 2018 to 11.8% (25/211) in 2020, while a rapid decline to 6.4% (21/330) in 2022 was observed. The resistance prevalence of NNRTIs was the highest (5.2%, 48/925), followed by INSTIs (1.9%, 18/925), NRTIs (1.2%, 11/925), and PIs (0.2%, 2/925). In 2020, the resistance prevalence of NRTIs, PIs, and NNRTIs increased the highest value, respectively, and then declined significantly. However, the resistance prevalence of INSTIs increased from 2.3% (3/128) in 2018 to 2.7% (4/147) in 2019, while a significant decline to 0.9% (1/109) in 2021 was observed, and then increased sharply to 2.1% (7/330) in 2022. Statistical analysis (**Table 4**) revealed that the resistance prevalence of NNRTIs, INSTIs, NRTIs, and PIs showed no increase or decrease in epidemic trend (*p* > 0.05); however, all kinds of PDRs presented a positive correlation (*r* > 0) except for NRTI-PDR (*r* < 0). Furthermore, **Figure 1** also indicated that the prevalence of CRF01_AE and CRF07_BC showed a decreasing and increasing trend, respectively, which was associated with the spread of HIV-1 PDR strains.

**Table 4:** Epidemic trend of main HIV-1 PDR circulating at the HSPs in Hebei province, China.

PDR	2018 (*n* = 128)	2019 (*n* = 147)	2020 (*n* = 211)	2021 (*n* = 109)	2022 (*n* = 330)	Chi-square		Chi-square for trend		Spearman	
						*χ* ^2^	*p*	*χ* ^2^ trend	*p*	*r*	*p*
Overall PDR	8	14	25	9	21 ^a^	5.051	0.282	0.258	0.621	0.020	0.540
PI	0	0	2	0	0	4.268	0.224	0.149	0.833	0.015	0.653
NRTI	0	1	5	1	4	3.489	0.440	0.582	0.472	−0.023	0.483
NNRTI	5	9	15	7	12	4.223	0.377	0.511	0.475	0.027	0.404
INSTI	3	4	3	1	7	1.521	0.836	0.121	0.754	0.010	0.765

First, the difference between different PDRs is analyzed using the chi-square (*χ* ^2^) test. Further analysis will be carried out using chi-square for trend when a *p*-value (*χ* ^2^ test) for HIV-1 PDR is less than 0.05. Lastly, we use the Spearman method to analyze the epidemic trend of HIV-1 PDRs with a *p*-value of 0.05 (*χ* ^2^ trend): *r* < 0 denotes a negative correlation, and *r* > 0 denotes a positive correlation. PDR, primary drug resistance; PI, proteinase inhibitor; NRTI, nucleoside reverse transcriptase inhibitor; NNRTI, non-nucleoside reverse transcriptase inhibitor; INSTI, integrase strand-transfer inhibitor. ^a^In 2022, two participants contained dual resistance to NNRTIs and INSTIs.

### 3.4 HIV-1 subtypes associated with resistant mutations

In total, 15 kinds of HIV-1 subtypes were found in this study. Of them, 12 recombinant forms and three simple subtypes were identified. CRF01_AE (49.1%, 454/925) and CRF07_BC (32.6%, 302/925) were the most frequent subtypes. The prevalence of URFs (6.2%, 57/925) was significant higher than that of subtype B, becoming the third largest subtype. As indicated in **Table 1**, 77 participants harboring the PDR were mainly distributed in CRF01_AE (44.2%, 34/77), CRF07_BC (41.6%, 32/77), CRF55_01B (6.5%, 5/77), B (3.9%, 3/77), CRF65_cpx (1.3%, 1/77), CRF68_01B (1.3%, 1/77), and URFs (1.3%, 1/77). All of the PI- and INSTI-DRMs were circulating in participants with CRF01_AE and CRF07_BC; however, NNRTI-DRMs were distributed in six subtypes (**Table 1**). There were no statistical differences in resistance to PIs, NRTIs, NNRTIs, and INSTIs between different HIV-1 subtypes, ages, occupations, nationalities, educational levels, infection routes, and sample sources. However, the distribution of PI-, NRTI-, NNRTI- and INSTI-DRMs in initial CD4 counts and marital status showed obvious statistical differences (*p* < 0.05). The resistance rate will decline with the increase of initial CD4 counts, which suggests that early detection and early treatment can significantly reduce the resistance rate and increase therapy effect.

### 3.5 Transmission networks of resistant HIV-1 strains

**Figure 2:**
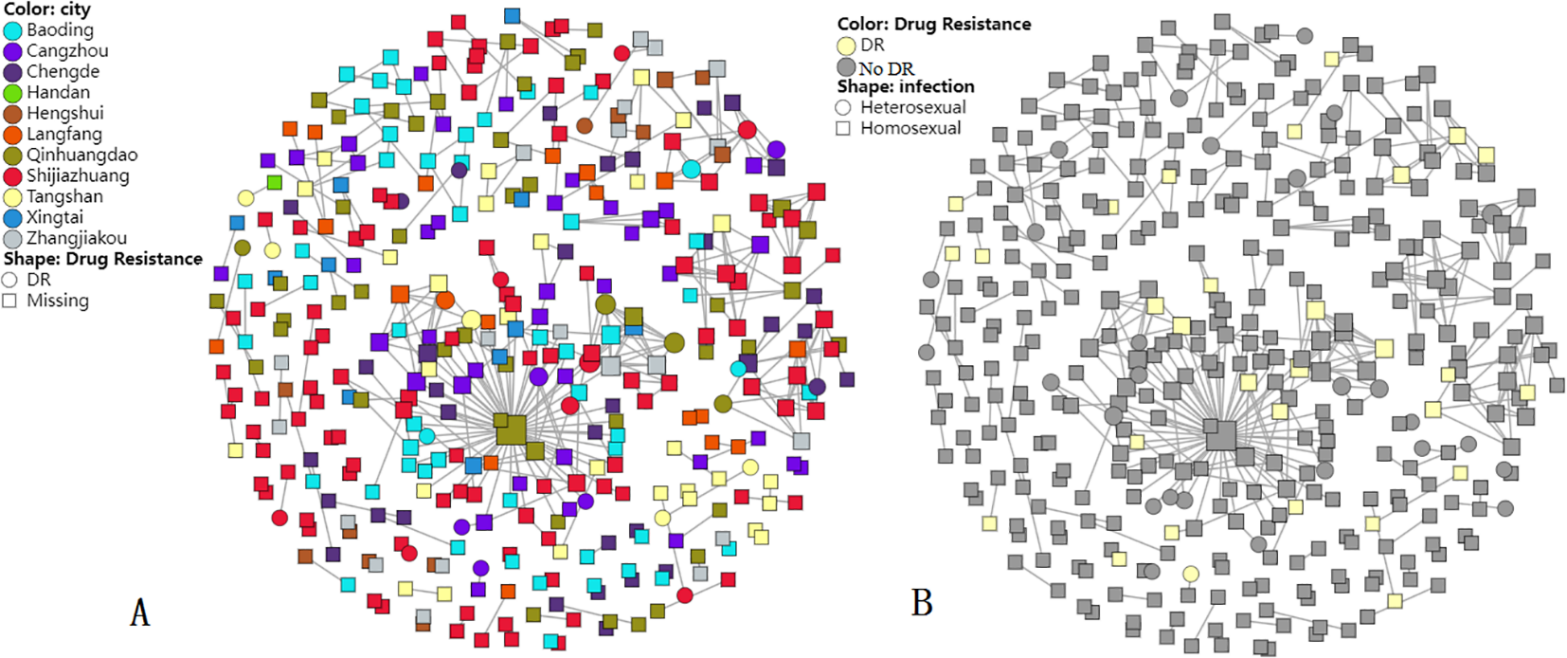
Molecular transmission networks of HIV-1-resistant strains based on HIV-1 near full-length pol gene sequences. **(A)** DR distribution in cities; **(B)** DR distribution in infection routes.

In total, 385 of 925 study sequences were detected within molecular transmission networks with a genetic distance threshold of 0.015. A total of 106 transmission clusters were constructed, and cluster size was 2 to 99 sequences (**Figure 2**). Of 106 transmission clusters, 16 contained DRMs. A total of 30 resistant sequences were circulating in 16 networks with HIV-1 PDR, accounting for 62.5% of 77 total participants with DRMs. Among 30 participants with DRMs, NNRTI-DRMs were the most common mutations (*n* = 17), namely, E138EG/K (*n* = 6), K103N (*n* = 4), Y181C (*n* = 1), Y188L (*n* = 1), L100L (*n* = 1), A98G (*n* = 2), Y318YF (*n* = 1), and G190GE (*n* = 1), followed by 9 INSTI-DRMs [G163G/R/S (*n* = 5), S153SF (*n* = 2), T66A (*n* = 1), Y143S (*n* = 1)], 3 NRTI-DRMs [T215TI/S (*n* = 2) and L74LI (*n* = 1)], and 2 PI-DRMs [F53L/I54L (*n* = 1) and N88NT/L90LM (*n* = 1)].

Most (92.7%) of the 385 sequences included in clusters were infected through MSM, followed by heterosexual contact (7.3%). Furthermore, sequences circulating in molecular transmission networks were from all 11 cities of Hebei province. In particular, the largest cluster harbored 99 CRF_07 BC sequences, and 11 sequences had DRMs in this cluster. The largest cluster included sequences from nine cities, forming an extensive resistant transmission network. Within the largest cluster, DRM sites included G163 (*n* = 3), Y143 (*n* = 1), K103 (*n* = 4), E138 (*n* = 1), Y188 (*n* = 1), L100 (*n* = 1), T215 (*n* = 1), and F53/I54 (*n* = 1), and one participant from Qinhuangdao had 58 links.

### 3.6 Factors related to HIV-1 PDR

**Table 5** lists 12 potential risk factors associated with PDR to PIs, NRTIs, NNRTIs, and INSTIs. Of these 12 factors, subtype and sample source were clearly associated with HIV-1 PDR (*p* < 0.05). Compared with participants with CRF01_AE, those who had CRF07_BC had 1.79 times greater odds of PDR [odds ratio (OR) 1.79, 95% CI 1.021–3.168]. Compared to participants with VBD, those with VCT had 0.27 times greater odds of PDR (OR 0.27, 95% CI 1.021–3.168).

**Table 5:** Factors related to PDR among treatment-naive HIV-1 individuals.

Factors	DR rate % (*N*)	Crude OR (95% CI)	*p*-value	Adjusted OR (95% CI)	*p*-value
Gender					
Male	8.5 (77)				
Female	0 (0)		0.998		
Age					
15–24	11.5 (22)				
25–49	8.69 (48)	0.732 (0.429–1.247)	0.251	0.832 (0.454–1.523)	0.550
≥50	3.9 (7)	0.316 (0.132–0.706)	0.010	0.387 (0.138–1.086)	0.071
Marital status					
Married/cohabitation	7.7 (28)				
Unmarried	10.4 (45)	1.400 (0.854–2.294)	0.182	1.133 (0.619–2.075)	0.685
Divorced/widowed	3.2 (4)	0.396 (0.136–1.151)	0.089	0.372 (0.126–1.094)	0.072
Initial CD4 counts (cells/μL)					
≤200	8.8 (15)				
201–500	7.3 (36)	0.814 (0.434–1.528)	0.522		
>500	9.8 (26)	1.112 (0.571–2,167)	0.755		
Infection route					
Heterosexual	2.7 (2)				
MSM	8.8 (75)	3.437 (0.824–14.249)	0.090	3.309 (0.800–14.366)	0.097
Nationality					
Han	8.3 (72)				
Ethnic minority	8.9 (5)	1.085 (0.420–2.805)	0.866		
STD					
No	8.2 (63)				
Yes	9.1 (14)	1.124 (0.613–2.062)	0.706		
Sexual partner					
1	6.8 (14)				
≥2	8.8 (63)	1.308 (0.717–2.386)	0.381		
Educational level					
High school or below	7.9 (49)				
College or above	9.2 (28)	1.184 (0.728–1.925)	0.495		
Subtype					
CRF01-AE	6.5 (24)				
CRF07-BC	10.6 (32)	1.714 (0.986–2.978)	0.056	1.798 (1.021–3.168)	0.042
B	6.4 (3)	0.986 (0.285–3.408)	0.982	0.949 (0.270–3.343)	0.935
Others	8.8 (18)	1.392 (0.736–2.630)	0.309	1.321 (0.679–2.571	0.412
Region					
North Hebei	10.9 (16)				
East Hebei	7.1 (14)	0.630 (0.297–1.335)	0.228		
Middle Hebei	8.4 (45)	0.749 (0.410–1.367)	0.347		
South Hebei	4.4 (2)	0.381 (0.084–1.723)	0.210		
Sample source					
VBD	19.4 (6)				
Special survey	11.5 (12)	0.543 (0.185–1.593)	0.266	0.495 (0.163–1.498)	0.213
VCT	6.6 (38)	0.295 (0.114–0.762)	0.012	0.274 (1.021–3.168)	0.010
Visit test	9.8 (21)	0.451 (0.166–1.224)	0.118	0.421 (0.151–1.180)	0.100

## 4 Discussion

This study is a follow-up work of our previous publication. In our previous publication, all of the participants were only MSM who came from the HIV surveillance points (HSPs) in Hebei, China, and the sample size is small. We mainly analyzed HIV-1 subtypes and their distribution. Only the resistance rate to PIs, RTs, and INSTIs was described at the HSPs, and other data related to resistance were not analyzed (Lu et al., 2024). In our current work, we reported HIV-1 PDR to NNRTIs, NRTIs, PIs, and INSTIs among newly diagnosed treatment-naive HIV-1 individuals between 2018 and 2022, including each gene mutation point and its resistance level to different drugs, the distribution of mutation points, factors related to the resistance, transmission risk of HIV-1 resistance strains, and so on. This study revealed that the overall prevalence of HIV-1 resistance to four kinds of drugs was 8.3% in Hebei, significantly lower than those in some developed countries such as USA (18.9%) (McClung et al., 2022) and Germany (17.8%) (Melanie et al., 2020), and we think that this difference may be due to the difference in the prevalence of HAART therapy, medication compliance, risk behaviors, and economic level both here and abroad.

Moreover, the annual resistant rate was 5.0% from 2018 to 2022, suggesting a moderate resistant level (5.0%–15.0%) (Bennett et al., 2008). The PDR prevalence of all inhibitors showed no increase or decrease in epidemic trend, and all of the PDRs presented a positive correlation except for NRTI-PDR.

One country-level study (Liu et al., 2023) showed that the overall prevalence of HIV-1 PR-RT PDR increased from 4.4% in 2017–2018 to 6.9% in 2021–2022. The recent report (Ye et al., 2024) also identified that the overall PR-RT PDR increased significantly from 4.55% in 2018 to 6.46% in 2023 in China. Our study revealed that the overall prevalence of HIV-1 PR-RT PDR increased from 3.9% in 2018 to 4.2% in 2022, significantly lower than national-level reports (Liu et al., 2023; Ye et al., 2024). In 2020, the prevalence of HIV-1 PR-RT PDR reached the highest value, and then declined significantly, presenting a parabola shape between 2018 and 2022. This suggests that strict prevention and control measures have disrupted national AIDS free treatment program during the coronavirus disease (COVID)-19 epidemic and decreased HIV-1 patients’ medication compliance, resulting in a dramatic increase of resistant mutations. Moreover, the prevalence of HIV-1 PDR began to decrease with the process of HAART normalization. Moreover, the central treatment regimen used in Hebei is two NRTIs and one NNRTI, accounting for >75%, and in cases of therapeutic failure, a treatment regimen including two NRTIs and LPV/r is implemented (Lu et al., 2017; Lu et al., 2023). We think that PR-RT PDR prevalence is expected to increase with the increasing use of the above drugs and subsequent treatment failure.

In this study, the overall PDR prevalence of NNRTIs was the highest (5.2%), which is significantly lower than previous studies from China’s eight provincial-level administrative divisions (6.3%) (Chen et al., 2023) and most countries (≥10%) in Africa, America, and Asia (World Health Organization, 2021). E138EK/G (1.6%) and K103N (1.4%) were the most common mutation sites, leading to low-level resistance to NVP/ETR/RPV and high-level resistance to NVP/EFV, respectively. Over the past 5 years, we observed that the rates of NRTI-PDR (1.2%) and PI-PDR (0.2%) in Hebei appeared to be lower than the national overall level and WHO’s estimated regional PDR rates (World Health Organization, 2021) in Americas (NRTI PDR: 6.4%; PI PDR: 0.8%) and Southeast Asia (NRTI PDR: 3.1%; PI PDR: 0.4%). Although the prevalence of NRTI- and PI-PDR remains low, the above low resistance prevalence might be an uprising challenge as the most common use in first-line therapeutic regimens.

The prevalence of INSTI-DRMs was first analyzed in a large Hebei sample. The resistance prevalence of INSTIs was the highest (2.7%) in 2019, while a significant decline to 0.9% in 2021 was observed, and then rose again to 1.2% in 2022. In this study, the overall prevalence of INSTI-PDR was 1.9%, which was significantly lower than the results of Yunnan (5.7%) (Deng et al., 2019), Taiwan (5.3%) (Chang et al., 2016), and Guangxi (3.1%) (Yan et al., 2023), but higher than the results in other regions in China (Song et al., 2021; Ye et al., 2024) such as Beijing (0.62%) (Yu et al., 2022) and Jiangsu (1.7%) (Yin et al., 2021). The HIV Drug Resistance Database (HIV Drug Resistance Database, 2022) and a previous study (Max et al., 2007) showed that INSTI mutation points can apparently reduce INSTI sensitivity, frequently located in 66, 92, 118, 140, 143, 147, 148, and 155. Six INSTI mutation points found in our study, namely, 153, 163, 145, 148, 143, and 66, can lead to different levels of resistance (from low to high level) to INSTIs, including EVG (1.1%), RAL (1.1%), DTG (0.8), BIC (0.8%), and CAB (0.8%). A total of 66.7% (4/6) were identified as major mutations (P145PS, Q148QH, Y143S, and T66A) associated with EVG and RAL.

Historically, WHO issued guidelines and recommended DTG-based ART as the first-line treatment in adults and adolescents in 2018 (World Health Organization, 2019). Until 2021, INSTIs have not been adopted as the preferred second-line ART use by adults, pregnant women, adolescents, and children under Chinese AIDS treatment guidelines (Acquired Immunodeficiency Syndrome and Hepatitis C Professional Group et al., 2022). However, all patients have to take the INSTIs medication at their own expense in Hebei, and even the whole of China. The occurrence of INSTI-DRMs, especially major mutations, suggests that the effective use of INSTIs will face an unprecedented severe challenge in the future. In particular, DTG will be conditionally used as a component of free ART according to the manual for national free anti-AIDS treatment drugs (2023 edition) issued by China in June 2023 (Chinese Center for Disease Control and Prevention, Center for STD/AIDS Prevention and Control, 2023), which can significantly reduce costs of patients receiving INSTIs ART. Therefore, although INSTIs have high potency, good tolerability, and high genetic barrier to resistance (Chinese Center for Disease Control and Prevention, Center for STD/AIDS Prevention and Control, 2023; Wong et al., 2016), the prevalence of INSTI-PDR will be expected to increase as observed for PI-, NRTI-, and NNRTI-PDR in the future with the increasing use of INSTIs nationwide.

Furthermore, 30 resistant sequences were circulating in 16 resistant networks, accounting for 62.5% of 77 total participants with DRMs. Within molecular networks, DRMs circulating in networks included resistant mutations to NNRTIs (E138EG, K103N, Y181C, Y188L, L100L, A98G, Y318YF, and G190GE), INSTIs (G163G/R/S, S153SF, T66A, and Y143S), NRTIs (T215TI/S and L74LI), and PIs (F53L/I54L and N88NT/L90LM), which suggests that HIV-1 PDR strains to NVP, EFV, EVG, RAL, and so on were in the sexual contact population especially in MSM (Yuan et al., 2022) infected with the predominant subtypes CRF07_BC and CRF01_AE. Moreover, compared with participants with CRF01_AE, those who had CRF07_BC had 1.79 times greater odds of PDR. Compared to participants with VBD, those with VCT had 0.27 times greater odds of PDR. The largest cluster (CRF07_BC) identified that participants from Qinhuangdao had a larger transmission risk than those from other cities, suggesting that we should also pay attention to areas with less HIV infections besides cities with more infections.

There is a limitation in this study: we only analyzed HIV-1 drug resistance mutations at the gene level due to lack of participants’ clinical data. In the future, we will strengthen *in vitro* resistance studies together with the hospital, which is responsible for treating AIDS in order to demonstrate *in vitro* “resistance” results to correlate to the resistance mutants.

## 5 Conclusions

In summary, the overall prevalence of HIV-1 PDR in Hebei is high, belonging to a moderate resistant level (5.0%–15.0%). In particular, the circulation of INSTI-DRMs provides an unprecedented severe challenge for the effective use of INSTIs in the future. Therefore, it is necessary for us to strengthen the effective surveillance of PDR among treatment-naive patients, and we should adjust the HAART plan according to the results of PDR surveillance.

## Data Availability

The original contributions presented in the study are publicly available. This data can be found here: partial HIV-1 sequences identified in this study have been deposited into GenBank with the accession numbers: ON007374, OR496590, OR400997, OP390172-OP390174, OP169133-OP169134, OK392124-OK392125, OQ513731-OQ513733, OP921950-OP921952, PP212966-PP212967.
